# The evolving Japanese encephalitis situation in Australia and implications for travel medicine

**DOI:** 10.1093/jtm/taad029

**Published:** 2023-03-03

**Authors:** Sarah L McGuinness, Colleen L Lau, Karin Leder

**Affiliations:** School of Public Health and Preventive Medicine, Monash University, Melbourne, Australia; Department of Infectious Diseases, The Alfred Hospital, Melbourne, Australia; School of Public Health, The University of Queensland, Brisbane, Australia; School of Public Health and Preventive Medicine, Monash University, Melbourne, Australia; Victorian Infectious Diseases Service, Royal Melbourne Hospital at the Peter Doherty Institute for Infection and Immunity, Melbourne, Australia

**Keywords:** surveillance, vaccination, emerging risks, travellers, Australia, Japanese encephalitis

## Abstract

The recent emergence of Japanese encephalitis in south-eastern Australia highlights the changing epidemiology of this important disease and the need for integrated surveillance to inform risk-based discussions and vaccination advice for travellers and endemic populations.

## Background

Japanese encephalitis virus (JEV) is the leading cause of viral encephalitis in Asia, causing ⁓100 000 cases and 25 000 deaths annually.^1^ JEV is transmitted by *Culex* mosquitoes in an enzootic cycle involving waterbirds (reservoir hosts) and/or pigs (amplifying hosts); humans are incidental dead-end hosts. Whilst most human infections are asymptomatic, severe disease occurs in ⁓1 per 250 infections and may rapidly progress to encephalitis with an estimated case fatality rate of 20–30%.^2^ Amongst survivors, ⁓30–50% have long-term neurologic sequelae.^3^ No specific treatment exists, but supportive care improves outcomes.

In early 2022, an outbreak of JEV in south-eastern Australia was initially detected through JEV testing of stillborn and weak piglets at piggeries, with subsequent detection of human cases across five Australian States and Territories.^4^ In March 2022, the outbreak was reported to the World Health Organization (WHO) and a Communicable Disease Incident of National Significance was declared.^5^ A resident of the Tiwi Islands who died from JEV infection in March 2021 was subsequently recognized as a sentinel case.^6^ As of February 2023, 46 human cases of JEV and seven deaths have been reported for this outbreak (Supplementary Table 1). Recent detection of JEV in piggeries, mosquitoes and sentinel chickens in the Murray River region, along with three human cases for the 2022–2023 summer season strongly suggests ongoing transmission in south-eastern Australia.

## Epidemiology of JE in Australia

In Australia, Japanese encephalitis (JE) is a nationally notifiable disease in humans and animals. Prior to 2021, the southern limit of JEV was the far north of Australia, with the only locally transmitted human cases reported in 1995 (three cases) and 1998 (two cases) from the Torres Strait Islands and Cape York Peninsula (Supplementary Table 2A).^5^ Subsequent animal serosurveillance in the Torres Strait has revealed seroreactivity to JEV in most years, but this was thought to represent seasonal incursions without establishment of endemic transmission on mainland Australia.^6^ Between 2012 and 2020, only 14 human JEV cases were notified to Australia’s National Notifiable Disease Surveillance System, with all acquiring their infection overseas.^7^

**Figure 1:**
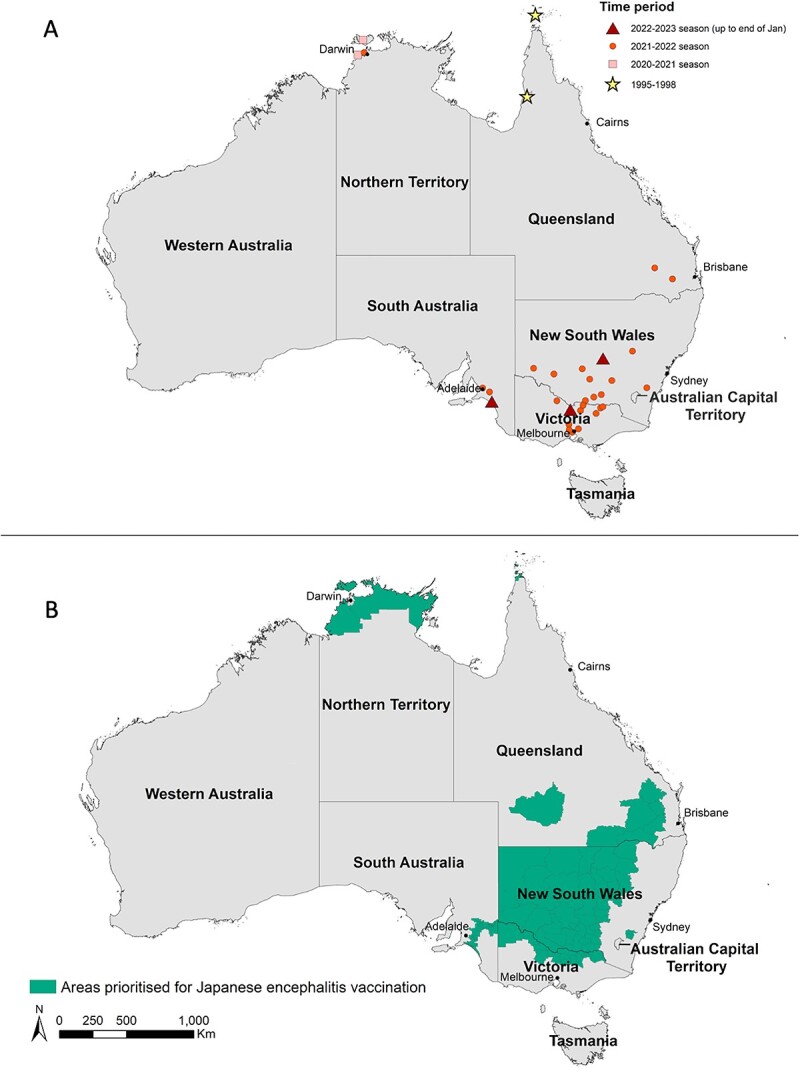
Panel A shows residential location for 40 of 46 human JEV cases in the current (2021–2023) outbreak and five of five cases in historical JEV outbreaks (1995 and 1998) in Australia. The location of symbols corresponds to the Local Government Area of residence of cases and may not reflect place of exposure. More than one cases was reported for some LGAs. Panel B shows areas prioritized for JE vaccination as of 31 January 2023. See Supplementary Tables 2A and B, and 3 for supporting data including links to the websites where the latest advice and official reports can be found.

Human cases and affected piggeries in the current outbreak have been widely distributed across four south-eastern Australian States [Victoria, New South Wales (NSW), South Australia and Queensland] and the Northern Territory where JEV has not previously been detected (Figure 1; Supplementary Table 2B). Revision to the outbreak case definition has led to two historical cases from 2021 being included in outbreak reporting. More cases have been reported in men than women and most cases have been reported in adults, with older adults most affected, consistent with an outbreak in an immunologically naïve population (Supplementary Figure 1).

Outbreak cases belong to Genotype IV JEV, previously found only within the Indonesian archipelago and (more recently) Papua New Guinea.^4^ Although unconfirmed, it is postulated that JEV GIV may have reached Australia via opportunistic dispersion of migratory birds to inland regions affected by heavy rainfall and flooding associated with La Niña weather patterns. Pigs appear to be an important amplifying host in the current outbreak.^4^ Recent modelling estimates that ⁓3% of Australia’s population live within 4.4 km (the purported flight range of *Culex annulirostis*, the primary Australian vector) of a piggery.^8^ A JEV serosurvey of volunteers living in and around five rural NSW towns from June–July 2022 found that 8.7% (80/917) had JEV antibodies.^9^

The geographical distribution of the current JEV outbreak mirrors that of previous outbreaks of other neurotropic flaviviruses (Murray Valley Encephalitis virus and West Nile virus Kunjin subtype) in south-eastern Australia associated with La Niña rainfall events.^4^ Whilst human cases in previous JEV outbreaks in north Australia (1995 and 1998) have occurred during the wet season months of Feb–May, human cases in the current outbreak have occurred over a longer transmission season (Nov–May). With climate change driving increases in the frequency, intensity and duration of extreme weather and climate events, including floods, and La Niña weather patterns continuing in 2023, endemic transmission is likely to continue.

## Prevention and Control

Prevention of JEV in humans centres around vaccination and mosquito bite avoidance. Several effective human vaccines are available, and two are licenced in Australia: the live-attenuated chimeric vaccine Imojev® (Sanofi Pasteur) and the inactivated Vero cell-derived vaccine JEspect® (Valneva; marketed as Ixiaro in Europe/North America).

Constraints to vaccine supply in Australia have influenced the public health rollout of JE vaccination, with eligibility for funded vaccination generally limited to people with occupational and/or relevant animal exposure risk (including people who work with pigs, mosquitoes or in a laboratory with JEV) and those who live or routinely work in affected Local Government Areas (LGAs) and are regularly outdoors for long periods (Supplementary Table 3). Following a recent evidence review, the Australian Technical Advisory Group on Immunization have indicated that intradermal (ID) administration of Imojev may be used as a dose-sparing strategy in settings where public health benefit outweighs potential risks, but highlighted that comparative data on immunogenicity and vaccine effectiveness are needed (https://www.health.gov.au/resources/publications/atagi-statement-on-the-intradermal-use-of-imojev-japanese-encephalitis-vaccine). Further studies are being conducted in Australia, including a field trial of ID Imojev in NSW (www.ncirs.org.au/japanese-encephalitis-vaccine-study).

## A One Health Approach

A One Health approach encompassing integration of data from human, animal, mosquito and environmental surveillance is integral to epidemiological understanding of JEV due to the enzootic nature of transmission. Human case surveillance data have limited ability to provide early signals of transmission due to the low rate of clinical disease amongst those infected (<1%) and limitations to diagnostic testing. Additionally, when JEV moves into previously non-endemic areas such as Australia, the low index of clinical suspicion may mean that early signals of local transmission are missed. Surveillance capacity varies across JE-endemic areas and previous estimates indicate that only ⁓10% of human cases are reported to the WHO.^10^ As vaccination programmes have become established in endemic areas for at-risk populations, human case numbers have declined but risk remains for unvaccinated individuals due to ongoing enzootic JEV circulation.^1^^,^^2^

Mosquito and animal surveillance play an important role in detecting JEV activity prior to the appearance of human cases, providing early warning signals that enable instigation of appropriate public health measures.^4^ In the Australian context, outbreak activity has occurred across a large geographical area with sparse human population, and serosurveillance of piggeries and mosquito trapping have had a major role in determining the extent of JEV transmission. Ongoing mosquito and animal surveillance is underway to monitor JEV activity and inform control strategies to limit transmission. An integrated One Health surveillance approach should also include environmental indicators (e.g. rainfall, flooding, vegetation, land use) related to transmission risk.

## Risk to Travellers

Current frameworks for assessing risks to travellers are generally based on country-level evidence of disease (presence/absence) and an assessment of individual trip characteristics associated with increased risk (e.g. extensive outdoor exposure). National and international guidelines generally recommend JE vaccination for travellers spending 1 month or more in risk areas during the transmission season and suggest that vaccination be considered for shorter-term travellers with additional risk factors (e.g. travel during transmission season, considerable outdoor activity planned, or staying in accommodation without air-conditioning, screens or bed nets; Supplementary Table 4).^3^ Previous estimates of JE risks in travellers have ranged from 1/400 000 per trip to <1 case per million travellers.^3^ Although the risk of JEV exposure is considered low for most travellers, the potentially devastating nature of the disease and favourable safety profile of available vaccines should prompt providers to discuss JE vaccination with all travellers to at-risk areas.^11^

Most travellers to Australia are at very low risk for JE, but ongoing risk and endemic transmission is likely, and the epidemiology is still evolving. US Centers for Disease Control and Prevention (CDC) guidance (https://wwwnc.cdc.gov/travel/destinations/traveler/none/australia) indicates that in Australia, JEV is mainly a concern around the Murray River (which flows from the Victorian Alps along the Victoria/NSW border and into South Australia) and Outer Torres Strait Islands (off the northern tip of Queensland). However, cases in the current outbreak have also been reported in southern Queensland, other parts of rural NSW, and in the Top End of the Northern Territory (Figure 1). We recommend that JE vaccination be discussed with all travellers who are moving to, frequently travelling to, or undertaking longer-term (e.g. ≥1 month) travel to these risk areas. We also recommend that JE vaccination be discussed with any shorter-term domestic or international traveller likely to visit risk areas from Nov-May, particularly if they are likely to be spending extensive time outdoors (e.g. camping). Additionally, we suggest that providers discuss mosquito bite avoidance measures with all travellers to Australia, given the presence of JEV and many other mosquito-borne infections such as Ross River virus, Murray Valley Encephalitis virus and West Nile virus/Kunjin virus.

## Conclusions

The reporting of locally transmitted human cases of JEV in two consecutive summers in south-eastern Australia likely reflects establishment of endemic transmission across a large part of subtropical and temperate Australia. Ongoing seasonal transmission is likely given ongoing La Niña conditions and increased frequency of extreme weather events driven by climate change. The epidemiological situation is still evolving in Australia, and risk areas may change over time.

In recent decades, documented JEV transmission has been limited to Asia, Papua New Guinea and Australia, but the epidemiology of the disease is changing, and many areas including the Pacific Islands, southern Europe, the United States and Africa remain receptive due to the presence of competent mosquito vectors and vertebrate hosts.^3^ The evolving situation in Australia has relevance to the possible threat that JEV may pose to these regions. An integrated, One Health approach to surveillance is needed to enable rapid detection of outbreak activity and define geographical areas of risk. Travel medicine practitioners should be aware of the changing epidemiology of JEV and discuss JEV with all travellers to endemic areas.

## Supplementary Material

JE_situation_Australia_JTM_perspective_supplementary_appendix_revised_clean_final_taad029

Supp_File_1_taad029

## Data Availability

The data that support the findings of this study are available in the Supplementary Materials.
