# Supplementary material for: The evolving Japanese encephalitis situation in Australia and implications for travel medicine
Source: J Travel Med. 2023 Mar 3;30(2):taad029. doi: 10.1093/jtm/taad029 (PMC10075061; doi:10.1093/jtm/taad029)
Supplement: JE_situation_Australia_JTM_perspective_supplementary_appendix_revised_clean_final_taad029 [file je_situation_australia_jtm_perspective_supplementary_appendix_revised_clean_final_taad029.docx]

**Supplementary Figure 1:** Age and sex distribution of JEV outbreak cases in Australia since 1 Jan 2021*

**
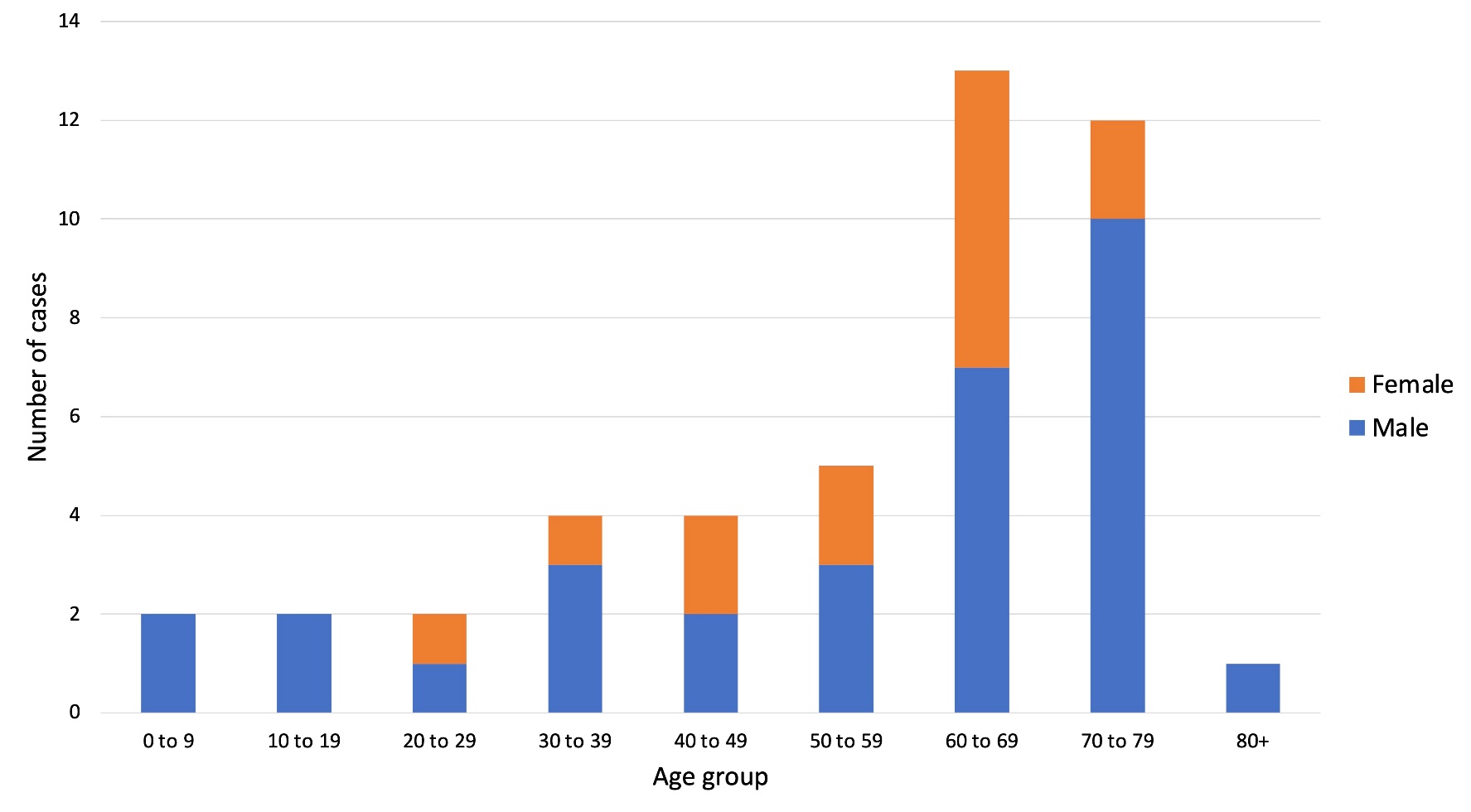
**

*****Age and sex information available for 45 out of 46 outbreak cases. Of these cases, 31/45 (69%) were male, 14/45 (31%) were female and 26/45 (58%) were aged 60 years or over. Data sourced from the National Notifiable Diseases Surveillance System (NNDSS) data visualisation tool, available at <https://www.health.gov.au/resources/apps-and-tools/national-notifiable-diseases-surveillance-system-nndss-data-visualisation-tool> (Accessed 21 Feb 2023)

**Supplementary Table 1:** Human cases of JEV notified in Australia since 1 Jan 2021^1^

| **State/territory** | **Total cases** | **Confirmed cases^2^** | **Probable cases^2^** | **Deaths** |
| --- | --- | --- | --- | --- |
| New South Wales | 14 | 14 | 0 | 2 |
| Northern Territory | 2 | 2 | 0 | 1 |
| Queensland | 5 | 2 | 3 | 1 |
| South Australia | 10 | 6 | 4 | 2 |
| Victoria | 15 | 12 | 3 | 1 |
| **Total** | **46** | **36** | **10** | **7** |

JEV, Japanese encephalitis virus

^1^Data from Australian Government website, last updated 13 Feb 2023, Available from: [https://www.health.gov.au/health-alerts/japanese-encephalitis-virus-jev/japanese-encephalitis-virus-jev](https://www.health.gov.au/health-alerts/japanese-encephalitis-virus-jev/japanese-encephalitis-virus-jev?language=en) (Accessed 21 Feb 2023). All human cases reported by the Department of Health have been verified by state reference laboratories listed under the Public Health Laboratory Network, see: <https://www.health.gov.au/committees-and-groups/phln/members>.

^2^Case definitions are set by the Communicable Diseases Network of Australia, and were last updated on 23 Jan 2023; full details are available from: [https://www.health.gov.au/resources/publications/japanese-encephalitis-virus-infection-surveillance-case-definition](https://www.health.gov.au/resources/publications/japanese-encephalitis-virus-infection-surveillance-case-definition?language=en) (Accessed 21 Feb 2023). Briefly, a confirmed case requires *laboratory definitive evidence*, defined as a) isolation of JEV by culture OR b) detection by nucleic acid testing (NAT) specific for JEV OR c) IgG seroconversion or a diagnostically significant increase in antibody level or a fourfold or greater rise in JEV-specific IgG titres with no history of recent JE vaccination OR d) detection of JEV-specific IgM in cerebrospinal fluid (CSF), without the detection of other flavivirus-specific IgM. A probable case requires *laboratory suggestive evidence*, defined as a) detection of JEV-specific IgM in CSF which is significantly greater than other flavivirus-specific IgM levels (if also detected)* OR b) detection of JEV-specific IgM in serum* with no history of recent JEV vaccination OR c) detection of JEV-specific IgG in CSF* in individuals with no recent history of recent JEV vaccination unless case also has encephalitis illness compatible with JEV infection in the absence of a known alternative cause. Testing should be performed at a laboratory with extensive experience in arboviral diagnostic testing.

*Either without detection of other flavivirus-specific IgM in serum/CSF or at levels significantly greater than that of other flavivirus-specific IgM levels (if also detected).

**Supplementary Table 2A:** Geographical location details and information sources for human JEV cases in previous north Australian outbreaks (1995 and 1998)

| **Local Government Area (place name)** | **State/ Territory** | **Transmission season** | | **Source of information** |
| --- | --- | --- | --- | --- |
|  |  | **1994-1995** | **1997-1998** |  |
| Torres Strait Islands (Badu Island) | QLD | 3 | 1 | Hanna JN, Ritchie SA, Phillips DA, Shield J, Bailey MC, Mackenzie JS, et al. An outbreak of Japanese encephalitis in the Torres Strait, Australia, 1995. Med J Aust. 1996;165(5):256-60. <https://doi.org/10.5694/j.1326-5377.1996.tb124960.x> |
| Carpentaria Shire (Mitchell River, Cape York) | QLD | 0 | 1 | Hanna JN, Ritchie SA, Phillips DA, Lee JM, Hills SL, van den Hurk AF, et al. Japanese encephalitis in north Queensland, Australia, 1998. Med J Aust. 1999;170(11):533-6. <https://doi.org/10.5694/j.1326-5377.1999.tb127878.x> |

**Supplementary Table 2B:** Geographical location details and information sources for human JEV cases in the current outbreak (2021-2023)

| **Local Government Area (place name)^a^** | **State/ Territory** | **Transmission season** | | | **Source of information** |
| --- | --- | --- | --- | --- | --- |
|  |  | **2020-21** | **2021-22** | **2022-23** |  |
| Balranald | NSW | 0 | 1 | 0 | <https://www.health.nsw.gov.au/Infectious/jev/Pages/default.aspx> |
| Berrigan | NSW | 0 | 1 | 0 | <https://www.health.nsw.gov.au/Infectious/jev/Pages/default.aspx> |
| Carrathool | NSW | 0 | 1 | 0 | <https://www.health.nsw.gov.au/Infectious/jev/Pages/default.aspx> |
| Dubbo | NSW | 0 | 1 | 0 | <https://www.health.nsw.gov.au/Infectious/jev/Pages/default.aspx> |
| Federation (Corowa) | NSW | 0 | 2 | 0 | <https://www.health.nsw.gov.au/Infectious/jev/Pages/default.aspx>  <https://www.corowafreepress.com.au/news/corowa-local-critical-japanese-encephalitis/> |
| Goulburn Mulwaree | NSW | 0 | 1 | 0 | <https://www.health.nsw.gov.au/Infectious/jev/Pages/default.aspx> |
| Griffith | NSW | 0 | 2 | 0 | <https://www.health.nsw.gov.au/Infectious/jev/Pages/default.aspx> |
| Lachlan | NSW | 0 | 0 | 1 | <https://www.health.nsw.gov.au/Infectious/jev/Pages/default.aspx> |
| Lockhart | NSW | 0 | 1 | 0 | <https://www.health.nsw.gov.au/Infectious/jev/Pages/default.aspx> |
| Temora | NSW | 0 | 1 | 0 | <https://www.health.nsw.gov.au/Infectious/jev/Pages/default.aspx> |
| Wentworth | NSW | 0 | 1 | 0 | <https://www.health.nsw.gov.au/Infectious/jev/Pages/default.aspx> |
| Unknown | NSW | 0 | 1 | 0 | <https://www.health.nsw.gov.au/Infectious/jev/Pages/default.aspx> |
| Campaspe | VIC | 0 | 0 | 1 | <https://www.health.vic.gov.au/infectious-diseases/local-government-areas-surveillance-report>  <https://www.abc.net.au/news/2022-02-28/japanese-encephalitis-warning-about-mosquito-borne-disease/100866726> |
| Gannawarra | VIC | 0 | 1 | 0 | <https://www.health.vic.gov.au/infectious-diseases/local-government-areas-surveillance-report> |
| Greater Shepparton | VIC | 0 | 1 | 0 | <https://www.health.vic.gov.au/infectious-diseases/local-government-areas-surveillance-report> |
| Hobson’s Bay | VIC | 0 | 1 | 0 | <https://www.health.vic.gov.au/infectious-diseases/local-government-areas-surveillance-report> |
| Indigo | VIC | 0 | 2 | 0 | <https://www.health.vic.gov.au/infectious-diseases/local-government-areas-surveillance-report> |
| Macedon ranges | VIC | 0 | 1 | 0 | <https://www.health.vic.gov.au/infectious-diseases/local-government-areas-surveillance-report> |
| Melton | VIC | 0 | 1 | 0 | <https://www.health.vic.gov.au/infectious-diseases/local-government-areas-surveillance-report> |
| Moira | VIC | 0 | 3 | 0 | <https://www.health.vic.gov.au/infectious-diseases/local-government-areas-surveillance-report> |
| Nilumbik | VIC | 0 | 1 | 0 | <https://www.health.vic.gov.au/infectious-diseases/local-government-areas-surveillance-report> |
| Wangaratta | VIC | 0 | 1 | 0 | <https://www.health.vic.gov.au/infectious-diseases/local-government-areas-surveillance-report> |
| Wodonga (Ebden) | VIC | 0 | 1 | 0 | <https://www.health.vic.gov.au/infectious-diseases/local-government-areas-surveillance-report>  <https://7news.com.au/lifestyle/how-a-smiley-four-month-old-boy-ended-up-in-hospital-after-contracting-the-japanese-mosquito-virus-c-6017341> |
| Wyndham | VIC | 0 | 1 | 0 | <https://www.health.vic.gov.au/infectious-diseases/local-government-areas-surveillance-report> |
| Tiwi Islands | NT | 1 | 0 | 0 | <https://nt.gov.au/wellbeing/health-conditions-treatments/viral/japanese-encephalitis>  Waller C, Tiemensma M, Currie BJ, et al. Japanese Encephalitis in Australia - A Sentinel Case. N Engl J Med. 2022;387(7):661-2. <https://doi.org/10.1056/NEJMc2207004> |
| Top End | NT | 1^b^ | 1 | 0 | <https://nt.gov.au/wellbeing/health-conditions-treatments/viral/japanese-encephalitis> |
| Riverland and Murray Mallee | SA | 0 | 6 | 1 | <https://www.adelaidenow.com.au/news/south-australia/four-more-cases-of-japanese-encephalitis-confirmed-including-one-person-who-died-this-month/news-story/d28c79a057c93a082efba56d0e4f9e3f>  <https://amp.abc.net.au/article/101804552> |
| Adelaide Hills | SA | 0 | 1 | 0 | <https://www.adelaidenow.com.au/news/south-australia/four-more-cases-of-japanese-encephalitis-confirmed-including-one-person-who-died-this-month/news-story/d28c79a057c93a082efba56d0e4f9e3f> |
| Darling Downs | QLD | 0 | 1 | 0 |  |
| Southeast Queensland | QLD | 0 | 1 | 0 | <https://www.brisbanetimes.com.au/national/queensland/anti-mosquito-measures-mobilise-to-head-off-japanese-encephalitis-threat-20220307-p5a2fq.html> |

NSW – New South Wales, QLD – Queensland, SA – South Australia, VIC – Victoria, NT – Northern Territory.

^a^Geographic location details represent cases’ Local Government Area (LGA) of residence and may not necessarily reflect place of exposure/infection. LGA data was not available for some outbreak cases (include 3 cases from QLD and 3 from SA); these cases have been excluded from this table and from Figure 1.

^b^This case represents a resident of Victoria (LGA unknown) who acquired infection while travelling in the Top End; this is the reason for the mismatch between the number of cases corresponding to the NT in Supplementary Table 1 (two cases) versus this table (three cases).

**Supplementary Table 3**: National, State and Territory-based eligibility criteria for funded JEV vaccination in Australia

| **Jurisdiction** | **Eligibility criteria** | **Website** |
| --- | --- | --- |
| National^1^ *(applies to all jurisdictions)* | People with occupational and/or relevant animal exposure risk, including:   - People who work at, reside at, or have a planned non-deferable visit to a piggery, including but not limited to farm workers and their families (including children aged 2 months and older) living at the piggery, transport workers, veterinarians and others involved in the care of pigs OR pork abattoir or pork rendering plant - Personnel who work directly with mosquitoes through their surveillance (field or laboratory based) or control and management, and indirectly through management of vertebrate mosquito-borne disease surveillance systems (e.g. sentinel animals) such as environmental health officers and workers (urban and remote) and entomologists. - All diagnostic and research laboratory workers who may be exposed to the virus, such as persons working with JEV cultures or mosquitoes with the potential to transmit JEV; as per the Australian Immunisation Handbook^2^ | <https://www.health.gov.au/health-alerts/japanese-encephalitis-virus-jev/japanese-encephalitis-virus-jev-vaccines> |
| Australian Capital Territory (ACT) | People who are engaged in the prolonged outdoor recovery efforts (clean up) of stagnant waters following floods (as a volunteer or through work related to flood response efforts) in any of the affected Local Government Areas (LGAs) in NSW^3^ or Victoria^4^ | <https://www.health.act.gov.au/jev> |
| New South Wales (NSW) | People aged 2 months or older who live or routinely work in any of the LGAs of high JEV concern^c^ **and**:   - spend significant time outdoors (four hours per day), for unavoidable work, recreation, education, or other essential activities, **or** - are living in temporary or flood-damaged accommodation (e.g. camps, tents, dwellings exposed to the external environment) that place them at increased risk of exposure to mosquitoes, **or** - are engaged in the prolonged outdoor recovery efforts (clean up) of stagnant waters following floods. | <https://www.health.nsw.gov.au/infectious/jev/pages/vaccination.aspx> |
| Northern Territory (NT) | People who live in a high-risk area^e^ which has:   - High mosquito activity, with limited or no control measures - JE positive pigs - Water birds - Past human cases | <https://nt.gov.au/wellbeing/health-conditions-treatments/viral/japanese-encephalitis> |
| Queensland | People belonging to the following at-risk groups:   - Pig doggers and hunters - People who live or work in the LGAs of Balonne, Goondiwindi, North Burnett, Quilpie, South Burnett, Western Downs or southwest area of Toowoomba Regional Council (surrounding and including Millmerran) **and** are at risk of JEV infection due to occupational or recreational outdoor activities undertaken near potentially productive mosquito habitat, such as areas near rivers, ponds and marshes, including flood zones and wherever there are bodies of standing water - People who live or work in the Torres Strait and/or Northern Peninsula Area of Cape York. | <http://conditions.health.qld.gov.au/HealthCondition/condition/14/217/83/japanese-encephalitis> |
| South Australia (SA) | The following groups are eligible for vaccination:   - people aged 2 months and older who have a primary residence in an eligible postcode^f^ **and** spend at least 4 hours per day outdoors most days - people aged 2 months and older who have a secondary residence affected by the River Murray flood in an eligible postcode **and** spend at least 4 hours per day outdoors most days. - people employed in a role located in an eligible postcode below **and** spend at least 4 hours per day outdoors as part of their occupation most days. | <https://www.sahealth.sa.gov.au/wps/wcm/connect/public+content/sa+health+internet/conditions/infectious+diseases/japanese+encephalitis/access+to+japanese+encephalitis+virus+vaccine> |
| Tasmania | Only people travelling to or residing on mainland Australia, or the Torres Strait and Tiwi Islands **and** who meet one of the following criteria are eligible for vaccination:   - - work at or reside at a piggery, including but not limited to farm workers and their families (including children aged 2 months and older), transport workers, veterinarians and others involved in the care of pigs   - work at or reside at a pork abattoir or pork rendering plant   - work directly with mosquitoes through their surveillance (field or laboratory based) or control and management, and indirectly through management of vertebrate mosquito-borne disease surveillance systems (e.g. sentinel animals) such as environmental health officers and workers and entomologists   - diagnostic and research laboratory workers who may be exposed to the virus, such as persons working with JE virus cultures or mosquitoes with the potential to transmit JE virus; as per the Australian Immunisation Handbook.   - living near a piggery with current or recent JEV activity   - feral pig hunters and doggers   - individuals living, working, or undertaking frequent recreational activities in regions, including aquatic habitats, determined to be at current or anticipated, near future JEV risk | <https://www.health.tas.gov.au/publications/japanese-encephalitis-virus> |
| Victoria | Anyone aged two months or older who lives or works in any of the high-risk local government areas (listed below) **and**:   - Spend significant time outdoors (four or more hours per day), for unavoidable work, recreation, education or other essential activities, **or** - Are living in temporary or flood damaged accommodation (e.g. camps, tents, dwellings exposed to the external environment) that place them at increased risk of mosquito bites, **or** - Are engaged in the prolonged outdoor recovery efforts (clean up) of stagnant waters following floods | <https://www.health.vic.gov.au/health-advisories/japanese-encephalitis-vaccination-update> |
| Western Australia | Eligible persons include:   - People living in the high-risk eligible postcodes (6740, 6743, 6770) in the Kimberley, excluding main towns - People working in an eligible postcode AND who spend at least 4 hours per day outdoors as part of their occupation most days. This excludes those working in the mining industry. - People who work directly with mosquitoes, and indirectly through sentinel animal surveillance systems in the Kimberley and Pilbara regions e.g. environmental health officers, entomologists, environmental consultants involved in mosquito management, rangers and park staff, and depot workers. - Diagnostic and research laboratory workers who are likely to handle live JEV. | <https://www.healthywa.wa.gov.au/Articles/J_M/Japanese-encephalitis> |

^1^National eligibility criteria are outlined on the Australian Government Department of Health Website, available here: <https://www.health.gov.au/health-alerts/japanese-encephalitis-virus-jev/japanese-encephalitis-virus-jev-vaccines> (Accessed 28 Jan 2023). All Australians that meet these criteria are eligible for free vaccination regardless of jurisdiction; additional State- and Territory-based eligibility criteria are outlined in subsequent rows.

^2^Australian Technical Advisory Group on Immunisation (ATAGI). Australian Immunisation Handbook, Australian Government Department of Health and Aged Care, Canberra, 2022, <https://immunisationhandbook.health.gov.au/> (Accessed 24 Jan 2023)

^3^List of affected LGAs in NSW is available on the NSW Government website: <https://www.health.nsw.gov.au/Infectious/jev/Pages/vaccination.aspx> (Accessed 24 Jan 2023)

^4^List of affected LGAs in Victoria is available on the Victorian Government website: <https://www.health.vic.gov.au/infectious-diseases/japanese-encephalitis-virus#vaccination> (Accessed 24 Jan 2023)

^5^Map of high risk areas in NT is available on the NT Government website: <https://nt.gov.au/wellbeing/health-conditions-treatments/viral/japanese-encephalitis> (Accessed 24 Jan 2023). High-risk areas correspond to the LGAs of Belyuen, Coomalie, East Arnhem, Litchfield, Tiwi Islands, Wagait, West Arnhem and West Daly.

^6^List of eligible postcodes is available on the South Australian Government website: <https://www.sahealth.sa.gov.au/wps/wcm/connect/public+content/sa+health+internet/conditions/infectious+diseases/japanese+encephalitis/access+to+japanese+encephalitis+virus+vaccine>

Abbreviations: JEV, Japanese encephalitis virus; LGA, Local Government Area; NSW, New South Wales; NT, Northern Territory

**Supplementary Table 4:** Japanese encephalitis (JE) Vaccine Recommendations for Travellers by National Health Authorities / Advisory bodies

| **Country** | **Year** | **National authority / Advisory body** | **JE vaccine recommended for** | **JE vaccine should be considered for** | **JE vaccine not recommended for** |
| --- | --- | --- | --- | --- | --- |
| Australia^1^ | 2022 | Australian Technical Advisory Group on Immunisation (ATAGI) | Travellers spending 1 month or more in endemic areas during the JE virus transmission season | Shorter-term (<1 month) travellers to endemic areas, particularly if   - travel is during the wet season - there may be ongoing travel to at-risk areas - There is considerable outdoor activity during the travel - The traveller is staying in accommodation without air-conditioning, screens or bed nets | n/a |
| Canada^2^ | 2019 | Committee to Advise on Tropical Medicine and Travel (CATMAT) | n/a^*^ | Adults at risk who desire vaccine-induced protection. Providers should discuss with the traveller the anticipated benefits and harms (including financial costs) associated with JEV to help the traveller reach a decision that is consistent with their own values and preferences. | n/a |
| UK^3^ | 2018 | Public Health England | Travellers staying a month or longer in endemic areas during the transmission season, especially if travel will include rural areas. | Shorter-term (<1 month) travellers to endemic areas if the risk is considered sufficient (for example, those spending a short period of time in rice fields where the mosquito vector breeds, or close to pig farming) | n/a |
| USA^4^ | 2019 | Centers for Disease Control (CDC) Advisory Committee on Immunization Practices (ACIP) | Persons moving to a JE-endemic country to take up residence, longer-term (≥ 1 month) travellers to JE-endemic areas, and frequent travellers to JE-endemic areas | Shorter-term (<1 month) travellers with an increased risk for JE on the basis of planned duration, season, location, activities and accommodations and for travellers to JE-endemic areas who are uncertain about their specific travel duration, destinations or activities | Travellers with very low risk itineraries (e.g. limited to urban areas/outside of transmission season) |
| Global^5^ | 2015 | World Health Organization | Travellers to endemic areas with extensive outdoor exposure during the transmission season, migrants to JE-endemic areas | n/a | n/a |

^*^CATMAT suggests that JE vaccine not be routinely used for travel to endemic areas (conditional recommendation)

^1^Australian Technical Advisory Group on Immunisation (ATAGI). Japanese encephalitis Australian Immunisation Handbook, Australian Government Department of Health and Aged Care, Canberra, 2022, <https://immunisationhandbook.health.gov.au/contents/vaccine-preventable-diseases/japanese-encephalitis> (Accessed 24 Jan 2023)

^2^Committee to Advise on Tropical Medicine and Travel (CATMAT). Statement on prevention of Japanese encephalitis. Public Health Agency of Canada, 2019. Available from: <https://www.canada.ca/en/public-health/services/catmat/statement-prevention-japanese-encephalitis.html> (Accessed 24 Jan 2023)

^3^Public Health England, Immunisation Against Infectious Disease – The Green Book. Chapter 20, Japanese encephalitis. London, UK, 2018. Available from: <https://www.gov.uk/government/publications/japanese-encephalitis-the-green-book-chapter-20> (Accessed 24 Jan 2023).

^4^Hills SL et al. Japanese Encephalitis Vaccine: Recommendations of the Advisory Committee on Immunization Practices. MMWR Recomm Rep 2019; 68(No. RR-2):1-33. <https://doi.org/10.15585/mmwr.rr6802a1>

^5^World Health Organization (WHO). Japanese Encephalitis Vaccines: WHO position paper. Weekly Epidemiological Record 2015, 90(9):69-87. Available from: <https://www.who.int/publications/i/item/who-wer9009-69-88> (Accessed 24 Jan 2023)
