# Supplementary figures and images for: The evolving Japanese encephalitis situation in Australia and implications for travel medicine
Source: J Travel Med. 2023 Mar 3;30(2):taad029. doi: 10.1093/jtm/taad029 (PMC10075061; doi:10.1093/jtm/taad029)

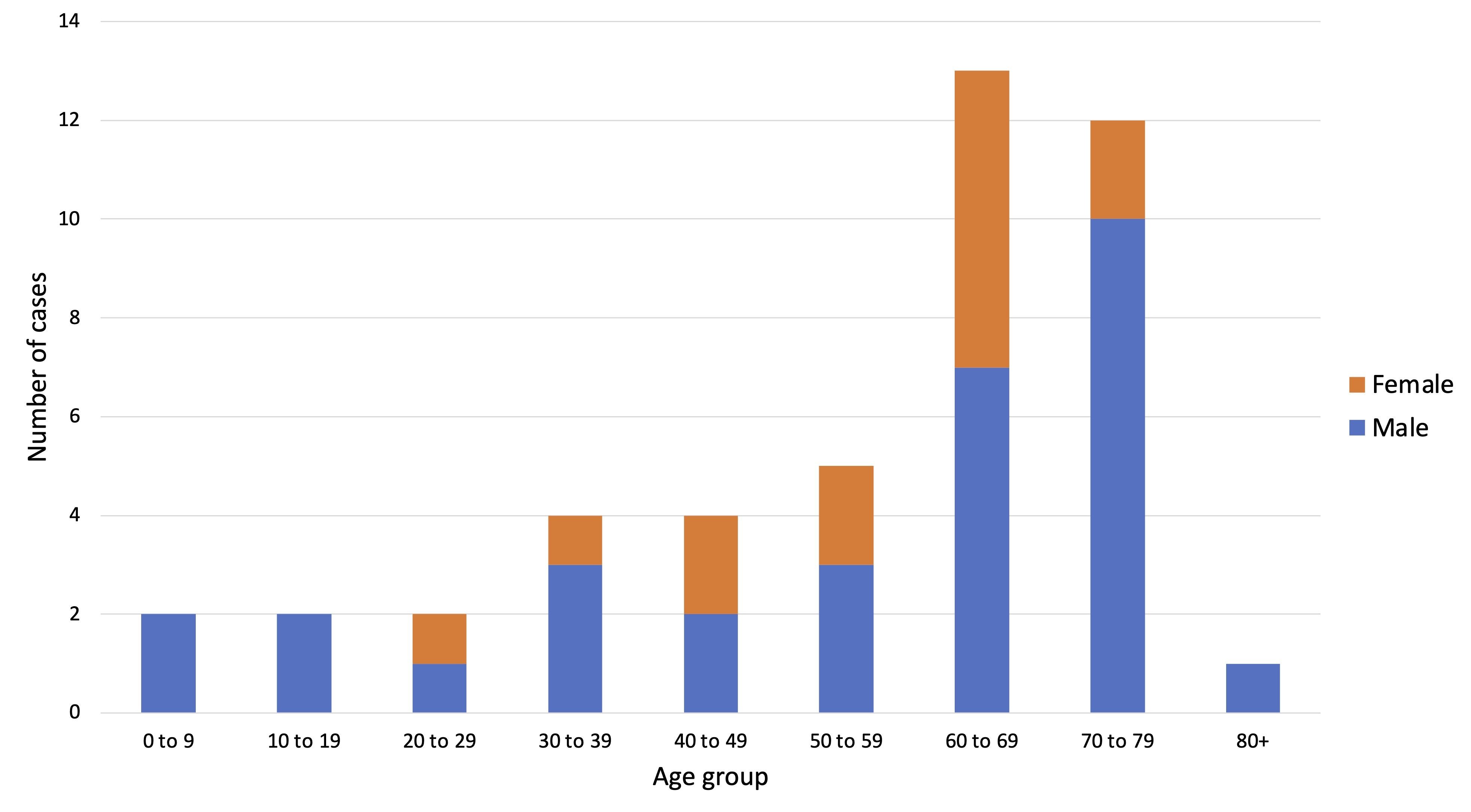

Supplement: Supp_File_1_taad029 [file supp_file_1_taad029.jpeg]
